# The Cognitive Mechanism of the Practice Effect of Time-Based Prospective Memory: The Role of Time Estimation

**DOI:** 10.3389/fpsyg.2019.02780

**Published:** 2019-12-06

**Authors:** Jiaqun Gan, Yunfei Guo

**Affiliations:** Faculty of Psychology, Southwest University, Chongqing, China

**Keywords:** time-based prospective memory, practice effect, training, time estimation, dynamic attention theory

## Abstract

Remembering to perform delayed intentions at a specific time point or period is referred to as time-based prospective memory (TBPM). The practice effect of TBPM is the phenomenon that TBPM performance improves *via* repeated PM training. In the present study, our main purpose was to explore the cognitive mechanism of the practice effect of TBPM, specifically the role of time estimation in the practice effect. We adopted a simple retrospective component of TBPM (pressing 1 key) in the present study, facilitating a closer look at the role of time estimation. In Experiment 1, the experimental group received 20 TBPM tasks training and some ongoing tasks training, while the control group only received some ongoing tasks training. We found that TBPM and time estimation abilities of experimental group were all better than those of control group. It proved that the practice effect of TBPM was closely related to the improvement of time estimation ability. In Experiment 2, we used time estimation training instead of TBPM training used in Experiment 1. The results of Experiment 2 were basically the same as those of Experiment 1. It further confirmed that time estimation played a key role in the practice effect of TBPM.

## Introduction

Prospective memory (PM) is the ability to remember to perform delayed intentions (Smith-Spark et al., 2016). There are two types of PM: time-based prospective memory (TBPM) and event-based prospective memory (EBPM). EBPM is executed when external cues appear, such as remembering to buy bread upon passing a bakery. However, rather than being precipitated by external cues, TBPM is executed at a specific time point or period, such as remembering to attend a professional class at 2:30 p.m. tomorrow. This study focuses on TBPM. TBPM consists of two components: prospective component and retrospective component. The prospective component of TBPM mainly relates to time estimation, while retrospective component of TBPM mainly involves the maintenance and extraction of intentional behavior, which is related to retrospective memory (Block and Zakay, 2006; Chen and Zhou, 2010). Compared with retrospective memory, PM directly impacts future events and quality of life. A survey found that more than half of our daily memory failures were the PM failures (Kvavilashvili et al., 2001). In light of the importance of PM, some studies have focused on how to improve its performance by behavior training. Brom and Kliegel (2014) further divided behavioral training into strategy training and cognitive processing training. Strategic training focuses on optimizing individuals’ strategies, while cognitive processing training focuses on improving individuals’ cognitive abilities. The present study explores the latter, specifically focusing on the phenomenon that PM performance improves with repeated PM training, which is defined as the practice effect of PM.

We first address whether the practice effect of TBPM exists. At present, some studies found that repeated TBPM tasks performed significantly better than occasional ones (Hu and Feng, 2013; Rose et al., 2015; Blondelle et al., 2016; Waldum et al., 2016). However, there is currently no research exploring its cognitive mechanism. The present study would focus on this point. From the perspective of the capabilities involved, the successful execution of TBPM relied mainly on time estimation and retrospective memory (Block and Zakay, 2006). If practice improved TBPM performance, it was likely to be closely related to the improvement of capabilities related to TBPM. The multiple processes theory held that EBPM could achieve automated extraction in specific situations (e.g., a mount of exercises) in order to prevent PM from consuming attention resources (McDaniel and Einstein, 2010), thus promoting PM performance. The view was also supported by some evidence (Rose et al., 2015). The retention and extraction of PM were related to retrospective memory, while TBPM and EBPM had the same retrospective component. Therefore, we inferred that automated processing of retrospective memory after training should increase with changes in the retrospective component of TBPM. However, temporal information processing requires self-initiated attention resources (Einstein et al., 1995), which made time estimation impossible to reach the state of automated processing, even after a large number of exercises. How did time estimation ability change when the practice effect of TBPM was observed?

Dynamic attention theory (DAT) was used to explain the changes of temporal information processing related to TBPM (Jones, 2006). DAT held that when individuals occasionally perceived specific time stimuli, their estimates of the time interval were imprecise, and their attention was relatively scattered. But when individuals perceived time stimuli periodically rather than intermittently, their estimates of the time interval became more accurate, with attention closer to the target time point (Large and Jones, 1999; Correa et al., 2006). According to DAT, periodic training for a specific TBPM task should increase individuals’ time estimation abilities, increasing their effectiveness at processing temporal information and potentially showing that time monitoring is increasingly concentrated near the point in time required by the task. In addition, repeated training for a specific TBPM task can also be regarded as training for a time estimation task to some degree. At present, some studies found that training for specific time intervals could significantly improve the accuracy of time estimation (Panagiotidi and Samartzi, 2013; Healy et al., 2015), as was consistent with DAT. Based on the above evidence and theoretical speculations, training for TBPM tasks likely increases time estimation accuracy, improving TBPM performance to some extent.

However, time estimation is an implicit process when we perform a TBPM task. We can only detect the changes of time estimation *via* indirect methods. On one hand, we can determine whether participants’ time estimation abilities are improved by comparing the time estimation performances between the control group (without TBPM training) and the experimental group (with TBPM training). On the other hand, changes in individuals’ time monitoring can also reflect changes in time estimation abilities to a certain extent. The test-wait-test-exit (TWTE) model held that when we performed TBPM tasks, the processes of “wait” and “test” repeatedly and alternately occurred (Harris and Wilkins, 1982). The “wait” could be seen as a process of time estimation while the “test” was a process of checking the time for external feedback. According to TWTE, changes in time monitoring could reflect changes in time estimation. Further, some studies found that individuals with high time estimation abilities tended to monitor time near the target time point when performing TBPM tasks (Labelle et al., 2009; Vanneste et al., 2016), indirectly validating the TWTE model. Therefore, we can judge individuals’ changes in time estimation by analyzing their changes of time monitoring when they perform TBPM tasks. If individuals’ time monitoring is closer to the time point required by the TBPM tasks, we can infer that the accuracy of their time estimation has improved.

The present study had two purposes. The first was to verify the existence of the practice effect of TBPM. We directly confirm this point by comparing the TBPM performances of the experimental group (after TBPM training) with the control group (no TBPM training). The second purpose was to explore the cognitive mechanism of the practice effect. We mainly focused on the role of time estimation in the practice effect of TBPM, which was also the main purpose of the present study. In addition, according to current evidence and theoretical speculation, both time estimation and retrospective memory might affect the practice effect of TBPM. However, the role of retrospective memory in the practice of PM was affected by the difficulty of the retrospective component. If the retrospective component was very easy, it was easy to maintain and extract the intention of PM in an automated process even without rehearsal and practice (McDaniel and Einstein, 2010). The present study simplified the retrospective component, in order to highlight the role of time estimation. In Experiment 1, we focused on whether both the TBPM performance and time estimation ability would significantly improve after training. If both improve simultaneously, we could infer that the practice effect of TBPM was closely related to individuals’ time estimation abilities. We further explored whether time estimation played a key role in the practice effect of TBPM in Experiment 2. During the training stage of Experiment 2, we specifically used time estimation training instead of TBPM training used in Experiment 1, in order to explore whether time estimation training would produce results that were similar to the practice effect of TBPM. Compared with previous studies, the present study was the first to explain the practice effect of TBPM from the changes of time estimation, which revealed the key role of time estimation in the habit formation related to TBPM.

## Experiment 1

In Experiment 1, we directly verified the existence of the practice effect of TBPM and further explored potential relationship between the practice effect and time estimation ability. The changes in time estimation ability could be reflected in many aspects. Firstly, the performance of the time estimation test could reflect participants’ time estimation abilities after training. Secondly, according to TWTE, time monitoring could indirectly influence the statuses of time estimation when participants performed the TBPM tasks. Also, the closer the time point of time monitoring was to the target time point, the more effective the time monitoring was (Vanneste et al., 2016), allowing us to adopt a time difference indicator in order to reflect the accuracy of time estimation. The time difference was the difference between the time point required by the TBPM task and the average time point of time monitoring. For example, the TBPM task required participants to press 1 key after 60 s. If they checked the time at 40 s and at 50 s, respectively, then the average time point of time monitoring was 45 s, with a time difference of 15 s. We predicted that participants had better TBPM performance and time estimation ability after repeated TBPM training.

### Method

#### Participants and Design

Fifty-seven undergraduate students participated in the experiment and received monetary compensation of 30 RMB. They were randomly assigned to the control group (25 participants, *M*_age_ = 20.40, SD = 1.66, 9 males) or to the experimental group (32 participants, *M*_age_ = 20.03, SD = 1.38, 10 males; due to a mistake about when to stop collecting data, we carelessly recruited more participants in the experimental group). All participants signed the informed consent form. The present study was approved by the Ethics Committee of Southwest University.

#### Procedure

The procedure started with instruction of ongoing task. Participants needed to practice 30 ongoing tasks with feedback. The materials for the ongoing task were 24 capital letters, comprising the English alphabet with F and J omitted. The ongoing task was a 1-back task, which required participants to compare the current letter with the one before it. If the two letters were the same, they were required to press the J key. Otherwise, they were required to press the F key. At the beginning of each ongoing task, a fixation (+) appeared in the center of the screen for 500 ms. Then, a capital letter appeared in the same position for up to 2,000 ms and disappeared when participants responded. Next, a blank screen of 500 ms would appear as a buffer and the trial ended. Their accuracy needed to exceed 0.9 before they could enter the formal experiment. The formal experiment included the training stage, the TBPM testing stage, and the time estimation testing stage. The training stage started with the instruction for the TBPM task. The TBPM task required participants to press 1 key once per minute. In addition, they were allowed to check the time by pressing the space key at any time, causing the time to appear at the bottom of the screen, disappearing after 1 s. There was no limit to the number of times participants pressed the space key. Participants in the experimental group were asked to perform both the ongoing task and the TBPM task, but participants in the control group were only asked to perform the ongoing task. In the training stage, both groups were asked to perform more than 700 ongoing tasks, while the experimental group was asked to perform 20 additional TBPM tasks. When time exceeded 65 s or participants pressed the 1 key, the program would initiate a break. Participants could decide how long the rest time was. After the break, the program would start from 0. Both groups rested 19 times during the training stage, which lasted about 20 min. In the TBPM testing stage, both groups were required to perform five TBPM tasks and more than 180 ongoing tasks. This stage lasted for about 5 min, during which time participants could take four breaks. The time estimation task required participants to reproduce a time interval of 60 s by pressing the space key twice. The filling stimulus of the time estimation task was similar to the ongoing task used in the present study. It started with a fixation (+) for 500 ms, then a capital letter in the same position, without requiring a response from participants, displayed between 300 and 2,000 ms. Finally, a blank screen would appear for 500 ms. When participants performed the time estimation task, the filling stimulus would appear cyclically. In the time estimation testing stage, there would be more than 120 filling stimuli in total. In addition, when estimating time, participants were asked not to use such strategies as counting, but rather to estimate time by feeling.

### Results and Discussion

**Figure 1:**
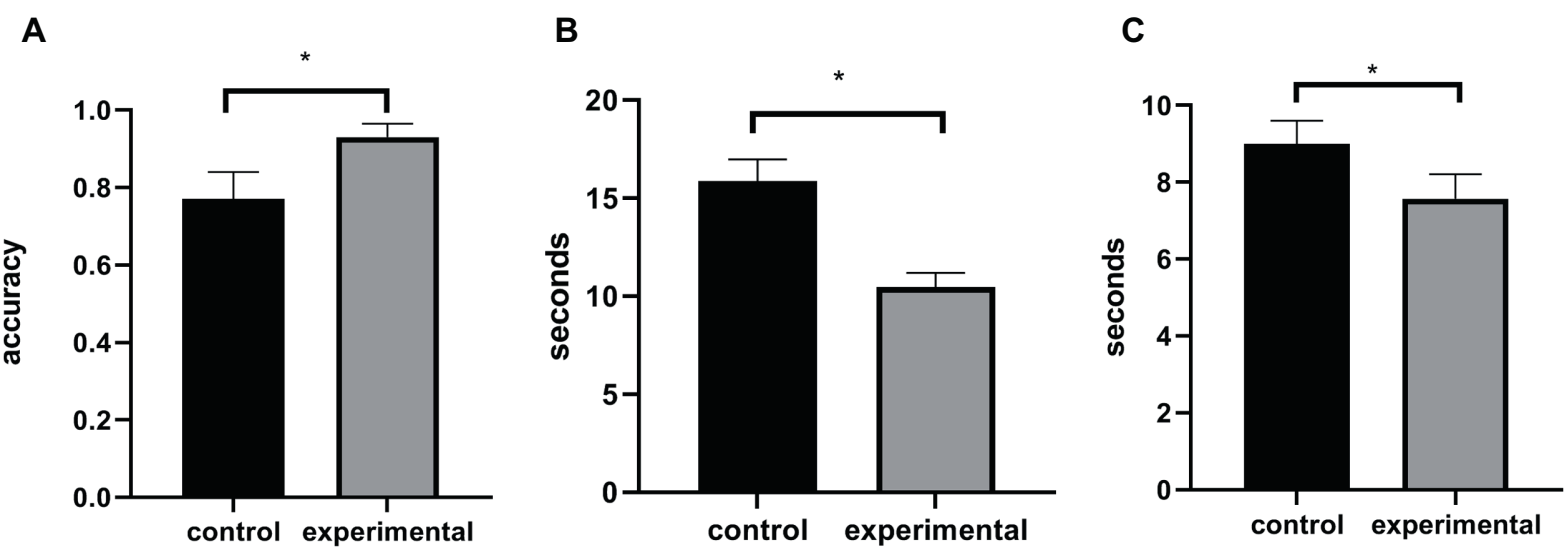
Time-based prospective memory (TBPM) performance **(A)**, time difference **(B)**, and time estimation performance **(C)** in Experiment 1. Control, control group; experimental, experimental group. Asterisks present statistically significant differences between control group and experimental group.

All of the indicators in Experiment 1 were analyzed by independent samples *t*-test. The results are also described in Figure 1. If participants pressed the 1 key within 5 s before or after 1 min (55–65 s), we determined that they had correctly performed the TBPM task. For TBPM performance, the results showed that the experimental group (*M* = 0.93, SD = 0.20) performed better than the control group (*M* = 0.77, SD = 0.35), *t*(55) = 2.24, *p* < 0.05, Cohen’s *d* = 0.60, which supported the practice effect of TBPM. For the accuracy and reaction time of the ongoing task, we found no difference between the experimental group (*M* = 0.95, SD = 0.03, *M* = 500 ms, SD = 65) and the control group (*M* = 0.94, SD = 0.03, *M* = 525 ms, SD = 60), *p’*s > 0.05, which indicated that the ongoing task was not affected by the practice effect. For the time difference, we found that the time difference in the experimental group (*M* = 10.45 s, SD = 4.17) was smaller than that in the control group (*M* = 15.85 s, SD = 5.66), *t*(55) = 4.15, *p* < 0.001, Cohen’s *d* = 1.11, which revealed that time monitoring was more effective in the experimental group. For the time estimation performance, we subtracted 60 s from the estimated time interval. For example, if the time interval between pressing the space key twice was 63 s, the difference was 3 s. We used this difference to represent the accuracy of time estimation. The results of time estimation showed that the difference between the experimental and control groups was marginally significant, *t*(55) = 1.87, *p* = 0.066, Cohen’s *d* = 0.5, and that the experimental group (*M* = 7.57 s, SD = 3.63) was smaller than the control group (*M* = 9.34 s, SD = 3.41). It revealed that the time estimation of the experimental group was more accurate than that of the control group.

In summary, we found that TBPM performance could be improved by repeated TBPM training, indicating the existence of the practice effect of TBPM. In addition, we also found that the practice effect did not interfere with the ongoing task, which showed that it did not occupy additional attention resources, as was consistent with DAT (Jones, 2006). Further, DAT proposed that periodic perception of specific time stimuli could improve the effectiveness of attention, possibly improving time estimation ability. Time difference and the time estimation performance could reflect changes of participants’ time estimation abilities. The results of these two indicators also showed that the collective time estimation ability of the experimental group was better than that of the control group, further supporting the prediction of DAT and the hypothesis of the present study.

## Experiment 2

In Experiment 1, we observed the practice effect of TBPM and a simultaneous improvement in time estimation performance. Combining the existing evidence and theoretical speculation, we could conclude that the practice effect of TBPM was closely related to improvement in time estimation ability. However, whether time estimation played a key role in the practice effect had not been fully demonstrated in Experiment 1. We would verify this point in Experiment 2. Specifically, we used time estimation training in Experiment 2 instead of the TBPM training that was used in Experiment 1. If it could produce an effect similar to the practice effect, then we could confirm that time estimation did play a major role in the practice effect.

### Method

#### Participants and Design

Fifty-four undergraduate students participated in the experiment and received monetary compensation of 30 RMB. They were randomly assigned to the control group (26 participants, *M*_age_ = 19.85, SD = 1.52, 8 males) or to the experimental group (28 participants, *M*_age_ = 19.75, SD = 1.27, 10 males). All participants signed the informed consent form.

#### Materials, Tasks, and Procedure

The materials and tasks of Experiment 2 were exactly the same as those in Experiment 1. The experimental procedure was also similar to that of Experiment 1, except that in the training stage of Experiment 2, the experimental group was required to practice the time estimation task while the control group was only asked to look at the screen (the material presented was the same in both groups). In both the experimental group and the control group during the training stage, there were more than 500 filling stimuli, which were the same as those in Experiment 1. Participants were not required to respond to the filling stimuli. The training stage lasted about 20 min.

### Results and Discussion

**Figure 2:**
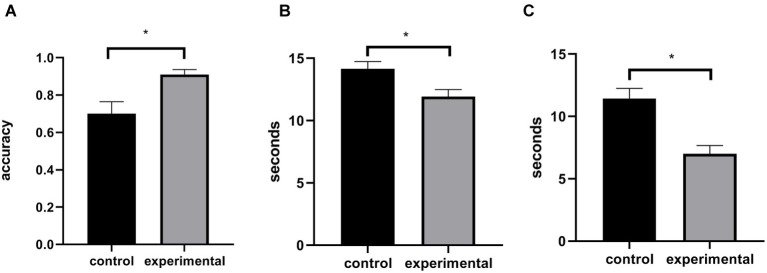
Time-based prospective memory (TBPM) performance **(A)**, time difference **(B)**, and time estimation performance **(C)** in Experiment 2. Control, control group; experimental, experimental group. Asterisks present statistically significant differences between control group and experimental group.

All of the indicators in the Experiment 2 were analyzed by independent samples *t*-test. The results are also described in Figure 2. If participants pressed the 1 key within 5 s before or after 1 min (55–65 s), we determined that they had correctly performed the TBPM task. For TBPM performance, we found that the estimation ability of the experimental group (*M* = 0.91, SD = 0.14) was better than the control group (*M* = 0.70, SD = 0.33), *t*(52) = 9.89, *p* < 0.05, Cohen’s *d* = 0.67, which showed that time estimation training could significantly improve TBPM performance. For the accuracy and reaction time on the ongoing task, we did not observe differences between the experimental group (*M* = 0.94, SD = 0.04, *M* = 557 ms, SD = 83) and the control group (*M* = 0.93, SD = 0.03, *M* = 595 ms, SD = 87), *p’*s > 0.05, suggesting that the improvement in TBPM performance did not interfere with the ongoing task. For the time difference and time estimation performance, the results showed that, compared with the control group (*M* = 14.15 s, SD = 3.73, *M* = 11.45 s, SD = 4.09), the experimental group (*M* = 11.93 s, SD = 3.71, *M* = 7.00 s, SD = 3.53) had smaller time difference and better time estimation performance, *t*(52) = 2.20, *p* < 0.05, Cohen’s *d* = 0.60, *t*(52) = 4.29, *p* < 0.001, Cohen’s *d* = 1.17, which revealed that time estimation training improved time estimation performance.

Overall, we trained the time estimation task in the training stage of Experiment 2. The results showed significant improvement in participants’ time estimation abilities, consistent with previous studies related to time estimation (Panagiotidi and Samartzi, 2013; Healy et al., 2015). Further, the TBPM testing stage revealed that TBPM performance could also improve by time estimation training, supporting our prediction. The results above indicated that time estimation indeed played a key role in the practice effect of TBPM.

## General Discussion

We focused on the practice effect of TBPM in the present study, which was essentially the plasticity of TBPM. However, the plasticity of TBPM could be triggered by many types of behavioral training (Rose et al., 2015). We focused only on the phenomenon that the TBPM performance could be improved by repetitive TBPM training. This phenomenon was called the practice effect of TBPM. The first concern of the present study was whether or not the practice effect of TBPM existed. The results of Experiment 1 showed that training improved TBPM performance, confirming the results of previous studies (Hu and Feng, 2013; Rose et al., 2015; Blondelle et al., 2016; Waldum et al., 2016). However, these four previous studies had obvious limitations in exploring the practice effect of TBPM, as was discussed before. We introduced a purer TBPM task that simplified the training strategy to only involve cognitive process training. TBPM performance also improved after training. The present study not only addressed these previous studies’ limitations, but also verified the reliability of their results. In addition, participants trained for only a short-term 20-min session. The results showed improvement in TBPM performance and indicated that the practice effect was clearly present when the time interval of TBPM was 1 min.

Our main purpose was to explore the cognitive mechanism of the practice effect of TBPM, specifically the role of time estimation in the practice effect. Once a TBPM task was successfully coded, its successful execution depended largely on time estimation ability and retrospective memory ability (Block and Zakay, 2006; Waldum and McDaniel, 2016). We speculated that both time estimation and retrospective memory might play a role in the practice effect. However, when retrospective memory related to the TBPM task was very simple, it was easy to reach a state of spontaneous extraction even without rehearsal and practice (Einstein and McDaniel, 2005; McDaniel and Einstein, 2010). In this case, retrospective memory was unlikely to benefit from repeated TBPM training, facilitating a closer look at the role of time estimation. Therefore, we defined a simple intention behavior (press the 1 key) in order to further highlight the role of time estimation in the practice effect. In Experiment 1, we found that when the practice effect of TBPM occurred, participants’ time estimation abilities significantly improved, indicating a close relationship between the two. In Experiment 2, we trained the participants for a time estimation task with a time interval of 1 min during the training stage, which could also significantly improve the TBPM performance. It further suggested that time estimation did play a key role in the practice effect of TBPM. In addition, some studies found that TBPM performance was related to participants’ attention input in temporal information processing. Additional attention to temporal information would improve TBPM performance (Voigt et al., 2011; Doyle et al., 2013; Vanneste et al., 2016). However, we found that participants’ attention to the TBPM task did not increase after training. Therefore, this possibility was excluded.

The key role of time estimation in the practice effect of TBPM task might be related to the characteristics of TBPM. When the retrospective component of PM was relatively simple, its intention could be maintained and extracted spontaneously without occupying attention resources (McDaniel and Einstein, 2010). In this case, the prospective component, not the retrospective component, determined whether PM could be successfully implemented. The prospective component of PM was mainly related to cue monitoring (Chen and Zhou, 2010). However, TBPM had no obvious external cues, and its cue monitoring could be seen as a process of time estimation in which participants could actively obtain feedback. Therefore, repetitive TBPM training could be regarded as repetitive time estimation training to some extent. Existing studies showed significantly improved time estimation ability following short-term training for estimating specific time intervals (Healy et al., 2015). In addition, some long-trained athletes had better timing ability in their own sports items (Chen et al., 2014; Vicario et al., 2017), which also indirectly proved our viewpoint. The results of the two indicators related to time estimation in the present study also verified the present study’s hypothesis. However, why did repeated TBPM training improve participants’ time estimation ability? In the implementation of the TBPM task, we not only have memory of intentional behavior, but also have memory of temporal information (Block and Zakay, 2006; Liverani et al., 2015). After estimating the interval repeatedly, temporal information experience could improve the accuracy of time estimation (Waldum and Sahakyan, 2013). In the present study, if participants realized that the time interval to complete 30 1-back tasks at their own speed was about 1 min after the training of 20 TBPM tasks, then they could use this information to help estimate time, thus improving the accuracy of time estimation.

This was the first study to systematically explore the cognitive mechanism of the practice effect of TBPM, which had important theoretical significance. According to previous evidence and theoretical speculation, we hypothesized that there were two main reasons contributing to the practice effect of TBPM: (1) time estimation ability had improved; (2) retrospective memory was in the state of spontaneous extraction. We set the difficulty of retrospective memory to be as easy as possible, which reduced the role of retrospective memory and highlighted the role of time estimation. The results also confirmed the key role of time estimation in the practice effect of TBPM. This verified some of our theoretical assumptions and laid a foundation for further theoretical models. However, there were some limitations in the present study. First, the major limitation in the present study was the absence of a baseline condition which would rule out alternative hypotheses related to inter-individual differences between the control group and the experimental group. Furthermore, the cognitive mechanisms for time estimation of different time intervals varied (Friedman, 1983). This phenomenon was also likely to occur in TBPM. In the present study, a time interval of 1 min was used to explore the practice effect of TBPM, and we concluded that time estimation played a key role in the practice effect. However, in terms of days or years, we were not sure whether time estimation would still play an equally important role. In addition, the difficulty of the retrospective component was set to be very easy, which reduced the role of retrospective memory. If the difficulty of the retrospective component increased, time estimation could not play a separate role in the practice effect of TBPM, and its role might be limited by retrospective memory. Finally, in the present study, the ongoing task was a relatively simple 1-back task, but the accuracy of time estimation was affected by the difficulty of the background task (Taatgen et al., 2007). Some studies suggest that time estimation accuracy would not improve significantly under the condition of high-difficulty background task even after training (Lio et al., 2006). Therefore, the difficulty of the ongoing task might also impact the practice effect of TBPM. Under a difficult ongoing task, the practice effect may not even occur. Despite the above limitations, we found that the practice effect of TBPM did exist under the conditions adopted in the present study, and that time estimation did play a key role in the practice effect.

## Data Availability Statement

All datasets generated for this study are included in the article/supplementary material.

## Ethics Statement

The studies involving human participants were reviewed and approved by Ethics Committee of Southwest University. The patients/participants provided their written informed consent to participate in this study.

## Author Contributions

JG performed the analysis. YG and JG performed the study and wrote the final report. YG was responsible for the design and planning of the study.

### Conflict of Interest

The authors declare that the research was conducted in the absence of any commercial or financial relationships that could be construed as a potential conflict of interest.
